# Fast sequence analysis based on diamond sampling

**DOI:** 10.1371/journal.pone.0198922

**Published:** 2018-06-28

**Authors:** Liangxin Gao, Wenzhen Bao, Hongbo Zhang, Chang-An Yuan, De-Shuang Huang

**Affiliations:** 1 Institute of Machine Learning and Systems Biology, School of Electronics and Information Engineering, Tongji University, Shanghai, China; 2 Science Computing and Intelligent Information Processing of GuangXi Higher Education Key Laboratory, Guangxi Teachers Education University, Nanning, Guangxi, China; Jilin University, CHINA

## Abstract

Both in DNA and protein contexts, an important method for modelling motifs is to utilize position weight matrix (PWM) in biological sequences. With the development of genome sequencing technology, the quantity of the sequence data is increasing explosively, so the faster searching algorithms which have the ability to meet the increasingly need are desired to develop. In this paper, we proposed a method for speeding up the searching process of candidate transcription factor binding sites (TFBS), and the users can be allowed to specify *p* threshold to get the desired trade-off between speed and sensitivity for a particular sequence analysis. Moreover, the proposed method can also be generalized to large-scale annotation and sequence projects.

## Introduction

Transcription factors (TFs) can suppress or activate gene expression by binding to specific DNA sites (TFBS),[1] and therefore play a central role in transcription regulation. Previous researches have concluded that TFs are inclined to bind to DNA sequences that follow specific patterns, called TF motifs.[2] Recently, the recognition of candidate TFBS on chromatin with a given TF motif has become a fundamental step to initiate the transcription of its target genes.

The existing candidate TFBS searching algorithms can be generally divided into the index-based algorithms and the online algorithms. The index-based algorithms commonly construct some special index structures, such as suffix trees[3, 4] or suffix arrays,[5] to accelerate the accessing of all the candidate’s locations on the target sequence. However, although these index structures could improve the searching efficiency, their construction costs in the time and space are remarkably huge. On the other hand, the traditional online approaches commonly scan a target sequence from left to right with a sliding window whose width is the same as the given TF motif, then report its candidate TFBS. Although these methods avoid the costs of constructing index structures, their time complexities still reach O(*mn*), where m and n are the lengths of the target sequences and the TF motif respectively. Recently, many technologies have been integrated into the traditional online algorithms, including matrix partitioning,[6, 7] Fast Fourier Transform[8], data compression[9] and other similar approaches to reduce the time complexity. In those methods, the most representative algorithm is lookahead approach[10] which resolves the problem of repeated matching by using partial thresholds that allow one to terminate the comparison of symbols when it is clear that no match will occur. However, it is unclear how efficient they can be in more challenging applications such as searching on high-throughput datasets or dealing with wider motif or less conservative TF motif. Additionally, in the TF motif modeling community, position weight matrix(PWM)[2], modeling the TF binding affinities with a matrix which describes the binding affinities as probability distributions over DNA alphabet, is one of the most commonly used TF models. And most of the candidate TFBS searching algorithms are based on this TF model, including naive algorithm, lookahead scoring algorithm, shift-add algorithm and so on.

Computationally, the given PWM can be regarded as the input features [11–13]. Motivated by this, the target sequence can be converted as a queried feature sets, if we split it into subsequences with a sliding window from left to right. Then the task of candidate TFBS searching can be abstracted as a classical k-Nearest Neighbor (KNN) problem, which is a widely studied formulation in the field of machine learning. With the formulation of candidate TFBS searching, there is no need to prepare index structures, in addition, it is anticipated that some advanced KNN technologies can be adopted to this formulation for obtaining high-quality solutions efficiently. In this paper, we will introduce a popular online search algorithm for quickly searching the significant matches. The inspiration come from the study of maximum all-pairs dot-product (MAD) searching problem which is the most complex part of the time complexity in the KNN technologies[14].

The key of KNN technologies is how to determine the similarity between samples which are represented by vectors with high-dimensional feature, i.e., **w** ∈ R^*d*^, for some large d in the real world. Many studies of real world calculate dot product as a criterion for judging similarity, hence, **v** ⋅ **w** is a usually useful measure of distance between **v** and **w**. Through MAD searching, the corresponding center point of every sample can be found, however, all-pairs dot-product takes a lot of time and in fact we only need to care about the large dot-product, not all-pairs dot-product. Diamond sampling algorithm[15] which is a new randomized approach is creatively promoted to solve the MAD problem by applying index sampling methods to avoid calculating all-pairs dot-product[16]. Designing a sampling procedure according to the relevant weights for the MAD problem is the key to the execution of diamond sampling algorithm.

The task of candidate TFBS searching is to find the max matching between position weight matrix and sequences. Using the idea of sampling according to the weights of diamond sampling algorithm, in the task of candidate TFBS searching, the original standard ordering which matches between position weight matrix and sequences in left-to-right order can be changed to a given permuted ordering[17]. The permuted ordering is given according to the matching failed expectation of every column in the position weight matrix and the corresponding expectation can be calculated through considering the distribution of background in the sequences, the information content of every column and so on.

In Results part, we demonstrated a series of experimental comparisons among the proposed algorithm and other current methods.

## Methods

### Notations and problem definition

The task of candidate TFBS searching is to find some appropriate subsequences for a given PWM in the sequences consisting of symbols in the four-nucleotide alphabet ℜ={A,C,G,T}. In some literatures, the position weight matrix which is a real valued n×|ℜ| matrix **M** = (**M**(*i*,*a*)) is also called position-specific scoring matrix, profile, and position weighted pattern. In this paper, we use PWM or pattern as for the abbreviation of position weight matrix for convenience. In the PWM, the coefficient (**M**(*i*,*a*) represents the probability of alphabet *a* at each position *i*. The length and the alphabet of the M can be represented *n* and ℜ, respectively. Table 1 is an example of pattern **M**.

**Table 1:** A position weight matrix in the nucleotide sequence databases.

*position*	1	2	3	4	5	6
**A**	0.14	-4.16	1.03	-4.16	0.58	-0.36
**C**	0.17	-2.31	-4.16	-4.16	-2.31	-1.32
**G**	-1.06	1.64	-2.32	-0.85	-1.06	1.12
**T**	0.12	-4.16	-2.64	1.18	0.07	-0.77

The pattern was obtained from the count matrix GATA-3 of JASPAR which was transformed into log-odds matrix using background distribution **q**_A_ = 0.278,**q**_C_ = 0.312, **q**_G_ = 0.212, **q**_T_ = 0.198. A pseudocount **q**_*s*_ was first added to the counts for each alphabet symbol s.

The given pattern **M** can match any segments **Seq** = *s*_1_*s*_2_…*s*_*n*_ where s_*i*_ represents a symbol in the alphabet ℜ. The significant of match is calculated by the match score G_*M*_(s) of s with corresponding **M**. The match score is defined as

$$G_{M}\left(S\right)=\sum_{i=1}^{n}M\left(i,s_{i}\right).$$ (1)

Given some biological sequences **Seq** = *s*_1_*s*_2_…*s*_*m*_ which consists of *m* symbols from the alphabet ℜ. For any subsequences *s*_*i*_*s*_*i*+1_…*s*_*i*+*n*-1_ (call a k-mer) of length *n* in the sequence **Seq**, the significant of match can be obtained with given the pattern **M**. The significant of match at location *i* in the **M** can be denoted as

$$g_{i}=G_{M}(s_{i}s_{i+1}\ldots s_{i+n-1})$$ (2)

In this paper, the problem of candidate TFBS searching can be described as follows: given a real-valued significance threshold *k*, the searching problem with threshold *k* is to find all location *i* in the sequence **Seq** such that *g*_*i*_ ≥ *k*. In addition to getting all location *i*, the values g_*i*_ should also be provided for many applications. Note that the traditional studied problem of exact string matching is just a special case of this search problem. The problem of exact string matching can be simply described as finding all the positions *i* which are the start position of the expectant string **s** = *s*_1_*s*_2_…*s*_*n*_ where all s_*i*_ is in the alphabet ℜ. We can generate a weight matrix **M** meeting the following conditions: **M**(*i*,*s*_*i*_) = 1, and **M**(*i*,*a*) = 0 if *a* ≠ *s*_*i*_, to solve easily the exact pattern search problem. The significant threshold *k* in this case can be explained for finding the exact positions where the alphabets of **s** match the corresponding alphabets of **Seq** in at least *k* positions. The problem of exact string matching can be solved easily when the significant threshold *k* be set to the same as the length of **s**.

### Preprocessing of pattern and significant threshold

If the background sequence distributions is different, the conservativeness of the corresponding PWM is also different, even if the pattern is the same as each other[18]. In order to make effective use of this nature, the values **M**(*i*,*a*) are in fact the log-odds scores of a probabilistic model of a signal to be detected against the background, such as finding putative binding sites of transcription factors in DNA. The signal model can be normally represented by a n×|ℜ| matrix **I** where **I**(*i*,*a*) is the probability of the alphabet symbol *a* occurring in the position *i*. There are many ways to obtain those probabilities such as from the corresponding empirically constructed count matrix and some of those probabilities may need to add pseudocounts to avoid logarithm with zero.

In order to facilitate the calculation, the background is usually constructed as a n×|ℜ| matrix B and every row of matrix **B** has the same probability vector which equal to the probability of background probability distribution at corresponding the alphabet symbols. Since the probabilities of every row is the same, we use **q**_*a*_ to denote the background probability distribution for a∈ℜ. Hence, **B**(*i*,a) = **q**_*a*_ for all *i*.

According to the log-odds score that consider the probability both in the signal model **M** and the background **B**, the match score between a subsequence and a matrix can be calculated as:

$$\begin{array}{l} Score(S)=log\prod_{i=1}^{n}\frac{I(i,S_{i})}{B(i,S_{i})}=\sum_{i=1}^{n}\log\frac{I(i,S_{i})}{B(i,S_{i})}\\ \;=\sum_{i=1}^{n}\log\frac{I(i,S_{i})}{q_{S_{i}}} \end{array}$$ (3)

Once the background **B** which is usually estimated from the sequence **S** is fixed, the model **I** can be translated into a position weighted matrix **M**:

$$M\left(i,a\right)=\log\frac{I\left(i,a\right)}{B(i,a)}$$ (4)

Then, the score computed by (1) equals the above *Score*(**S**).

The significance threshold *k* for the search is normally not easy to get, such that the standard statistical approach that use the p-values to control the confidence of the searching is more commonly used. When the p-value *p* is given, then the corresponding threshold is a value *k* = *k*(*p*) and the probability of subsequence **s** is *p* such that *G*_*M*_(s) ≥ *k* in the background distribution. In order to convert p-value *p* to the significance threshold *k* more quickly, we use a well-known pseudopolynomial time dynamic programming algorithm [10, 19, 20] to evaluate the corresponding *k = k*(*p*).

### The faster lookahead scoring algorithm

In this section, some theoretical analysis is firstly used to compare our fast searching algorithm with some well-known searching algorithms.

When **S**, **M** and *k* are given, the TFBS searching problem can be solved by evaluating w_i_ using (1) and (2) for each *i* = *1*,*2*,*…*,*m-n+1*, then reporting all location *i* and corresponding w_*i*_ such that *w*_*i*_ ≥ *k*. This primitive method is called as the naive algorithm (NA). For the time complexity of it, evaluating each w_*i*_ from (1) takes O(*n*) such that the total searching time approximates to O(*mn*), where *n* is width of the given pattern and *m* is the length of the given sequence **S**.

The main reason for which the low searching efficiency for the naïve algorithm is that the NA ignores the significant relationship between the score of segment w_*i*_ and the significance threshold *k*. When the NA algorithm process the subsequence, the NA algorithm does not end the matching process until it matches the end of the subsequence, even if it had been known some important information that the current subsequence cannot be matched successfully according to the currently matched information. In order to overcome the shortcoming of the NA algorithm, the lookahead scoring algorithm[10] is proposed as an improved algorithm of the NA algorithm, which utilize the significant relationship between w_i_ and the significance threshold *k* to speed up matching process. In order to guarantee each prefix of a candidate segment to decide quickly whether candidate segment match successfully, the lookahead scoring algorithm computes the intermediate score thresholds in advance. When the sequence **S**, the matrix **M** and the threshold *k* are given, although the score of the prefix of every candidate subsequences is fixed, the intermediate score thresholds cannot be directly computed by the score of the prefix candidate segment. Through the evaluation for the maximal score of the suffix of a candidate subsequent, the intermediate score thresholds can be defined for 1 ≤ *t* ≤ *n* as

$$P_{t}=\sum_{i=t}^{n}\underset{\alpha \in ℜ}{\max}\;M(i,\alpha )$$ (5)

Obviously, **p**_*t*_ is the maximum score of all possible matches for the suffix which starts at position *t+1* of any given candidate k-mer **S**_*i*_…**S**_*i*+*n*−1_. The segment ending at position t of candidate k-mer **S**_*i*_…**S**_*i*+*n*−1_ is the prefix and its match score can be computed as:

$$R_{t}=G_{M}(s_{i}\ldots s_{i+t‑1})=\sum_{j=1}^{t}M(j,s_{i+j-1})$$ (6)

When a segment is being matched at the position *t*, if **R**_t_+**P**_t_ is less than the matching significance threshold *k*, then we can draw a definite conclusion immediately that this k-mer cannot be matched successfully even if the matching score of suffix reach maximum. That reason of such decision can be interpreted as that the final match score is impossible for more than *k* for any possible k-mers which have the same prefix as the given sequence **Seq**.

When we have prior information about the possible match failure of currently matching segment, the matching process between sequences and model can be speeded up. In order to avoid the frequent calculation of intermediate score thresholds, all the intermediate thresholds can be calculated firstly as

$$T_{t}=k-P_{t}$$ (7)

And saved as an array for *t* = 1,…,*n*. The actual matching subsequence is generated by the sliding window with the length *n* on the given sequence, and the starting position of the sliding window increment by 1 for next match, for example, the subsequence **S**_*i*_…**S**_*i*+*n*−1_ is the current matching k-mer and the next match k-mer will be **S**_*i*+1_…**S**_*i*+*n*_. For the strategy of match, the current matching score is initialized to zero before matching the next subsequence and the accumulated score will add the value **M**(*t*,**S**_*i*+*t*_) of the matching alphabet at the position *t* only if the current accumulated score **R**_*t*_ is not less than **T**_*t*_. Once the above-mentioned conditions are not satisfied, the process of subsequence searching can be stopped at the current *i* and resumed next match at position *i+1*.

In the first section, we introduced that the task of candidate TFBS searching can be abstracted as a classical k-Nearest Neighbor (KNN) problem and the KNN problem can be viewed as a special case of maximum all-pairs dot-product (MAD) searching problem which can be solved by the diamond sampling algorithm. However, the diamond sampling algorithm provides an approximate result with the maximum probability rather than the precise result that we need in the task of candidate TFBS searching. Although the diamond sampling cannot be used directly to solve searching problem in the task of candidate TFBS searching, we can speed up the process of candidate TFBS searching with the weight sampling part of the diamond sampling algorithm.

Actually, the matching failed expectations at each position in the model is different from the task of candidate TFBS searching and those matching failed expectations can be viewed as some special weights to decide the order of the match. To speed up the task of candidate TFBS searching, we expect that the matching failed subsequence can be detected as early as possible by utilizing these special weights while the lookahead scoring algorithm ignores the information. Although the matching failed probability at each position can help us to speed up searching process indeed, it takes a very high cost to get the precise matching failed expectations. In this paper, we propose a further improved method based on the lookahead scoring algorithm, called the faster lookahead scoring algorithm (FLS), which utilize an approximate expectation to replace the precise matching failed expectations and the position with higher matching failed expectation should be matched firstly. The matching failed expectation at each position is influenced by many factors including the information content of each position in the PWM, the background distribution for the given sequences and so on. The approximate matching failed expectation at position *i* can be defined as

$$\begin{array}{l} E_{i}=\left|\underset{\alpha \in ℜ}{\min}\;M(i,a)\right|+\underset{\alpha \in ℜ}{\max}\;M(i,a)\\ \;-\sum_{\alpha \in ℜ}q_{a}M(i,a) \end{array}$$ (8)

Where **q** is the background distribution and **q**_*a*_ is the background probability of the alphabet *a*. The matching order can be defined as the decreasing order of the approximate matching failed expectation **E**_*i*_ for 1 ≤ *i* ≤ *n* in the matrix.

Due to the effect of permuted order for matching, the gap of maximum possible score and the actual score at position *i* will be widened by **E**_*i*_. So the partial score of matching subsequence, when evaluated in permuted order, will drop below the intermediate threshold **T**_*i*_ as much as possible on average. As a result, the failed matches in all subsequences will be detected as early as possible and the task of candidate TFBS searching will also take the minimal average time. Before the task of candidate TFBS searching, the intermediate score thresholds should be recalculated by the permuted order rather than by the original left-to-right order, and the permuted order is determined by decreasing all approximate matching failed expectations.

## Implementation

### Computing significance threshold

In our method, the significance threshold is not given directly but transformed from a specified probability threshold, called p-value, using quantile function. Its advantage is that the user can give a p-value to trade off the speed and sensitivity of the task TFBS candidate searching explicitly. Thus, the researcher can utilize the specified threshold to adjust the level of statistical significance for a particular analysis. A low p-value will reduce hits that are statistically very significant and lead to a high specific analysis. The advantage of this analysis is that the result can be relatively low false positives, but its disadvantage is that the result may miss much more distant sequence relationships. On the other hand, a high p-value is specified by user can analyze the particular sequence with high sensitivity. The calculation of significance threshold is difficult and a specific analysis usually requires a trade-off between relatively high speed and high sensitivity. Therefore, we utilize p-value to give a significance threshold indirectly in our method.

In order to calculate the quantile function more easily, some assumptions are declared as: the alphabet at each position in a random k-mer is independent and the background distribution is identical. The quantile function is not calculated directly, but by its inverse function, called the complementary cumulative distribution function (complementary CDF). On the other hand, the complementary CDF can be calculated by performing a summation over the probability mass function (PMF).[10]

We defines the segmental score as the random variable **X**, and the PMF of **X** by *f*(**x**). Then, if we give a particular segmental score *γ*, the p value can be defined as

$$G\left(\gamma \right)=\mathrm{Pr}\left\{X\geq \gamma \right\}=\sum_{x=\gamma }^{\infty }f\left(x\right)dx$$ (9)

Obviously, while the subsequence is assumed to be random in the task of TFBS candidate searching, the value of G(*γ*) is the probability of a score of k-mer, which the matching score is not less than *γ* in the given sequence.

For calculating the PMF, several methods have been proposed. In this paper, the adopted method is to compute the probability recursively with each column of the scoring matrix.[19, 21, 22] We defined a background frequency vector as

q=〈q(1),…q(|ℜ|)〉 (10)

Where ℜ is a set of possible alphabets. In our method, each position in a random k-mer is represented by a background frequency vector **q**. The background frequencies can be obtained by scanning the given sequence and counting number of various alphabets. Then, the PMF can be computed recursively, column by column:

$$\begin{array}{l} f^{\left(0\right)}\left(x\right)=\delta \left(x\right)\\ f^{\left(i\right)}\left(x\right)=\sum_{\alpha \in ℜ}q\left(\alpha \right)f^{\left(i-1\right)}\left(x-M(i,\alpha )\right)\\ \;i=1,\ldots ,m\\ f\left(x\right)=f^{\left(m\right)}\left(x\right) \end{array}$$ (11)

Where the function *δ*(*x*) is initialized to 0 for *x* ≠ 0 and 1.0 otherwise. However, the entire probability mass is usually initialized by score *x* = 0. Then, the revised version of the PMF, *f*^(*i*)^(*x*), is based on the previous PMF at each column *i*. In the process of computing PMF, simply (*x*−**M**(*i*,*a*)) is the score for all possible alphabet *a*. The desired PMF *f*(*x*) is generated at the final iteration.

Once we have the PMF *f*(*x*), the complementary CDF G(*γ*) can be computed by performing a summation over the PMF. Thus, the inverse function, G^-1^(*γ*), is expected as the quantile function. The quantile function generates a significance threshold T* = ⌈*G*^−1^(γ*)⌉ after given a *γ* threshold γ*. The computed G(*γ*) contain all possible k-mers score, such that the significance threshold can be calculated readily for any given thresholds.

### The overview of searching strategy

**Fig 1:**
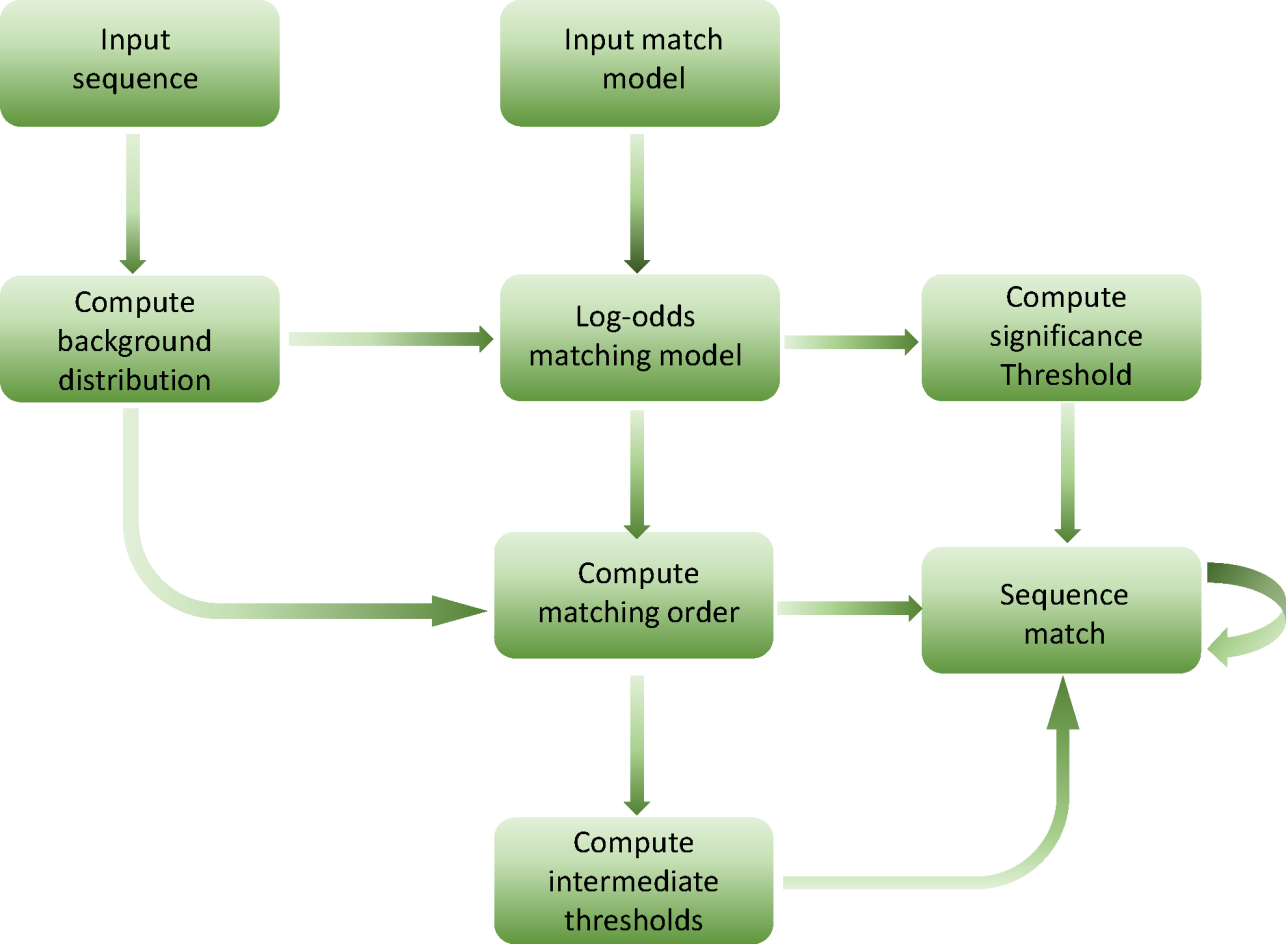
Overview of faster lookahead scoring algorithm with main steps.

The workflow of the proposed algorithm is presented in Fig 1. Since the length of a given sequence usually stand at hundreds of millions, as a saved-time technique, the corresponding background distribution can be calculated at the same time when the sequence is streaming to memory. In addition, the process of log-odds of matrix will produce error message when the matrix of model contain zeros. Thus, the probabilistic matrix needs to add a pseudocounts firstly at the position whose corresponding probability is zero before log-odds of matrix.

The process of matching between the given sequence and the model can be proceed after the significance threshold, matching order and the array of intermediate thresholds are all computed. In addition, the order of match is not left-to-right but following the matching order in (8).

## Results

In order to demonstrate the performance of our FLS (faster lookahead scoring algorithm), we implemented LS (lookahead scoring algorithm) and the well-known base-line algorithms NA (naive algorithm) as our contrast experiments from the original paper. The code of all experiments is written in C/C++ and the all tasks of candidate TFBS searching run for single-threaded. The running environment includes 3.2GHz Intel® Core™ i7-2600 processor with 3 gigabytes of main memory, running under Ubuntu 16.04, and gcc is the only compiler used in the experiments. We also repeat same experiments with 2.5GHz Intel® Xeon® CPU e5-2650 v2, and get essentially similar results which slight differences explained by different cache memory size.

### Datasets

With the development of gene sequencing, the size of the sequence database is increased explosively.[13, 22–24] In order to analyze these large number of sequence data, the efficient mathematical methods and computer algorithms used in the task of candidate TFBS searching need simple, logical and self-consistent. Information theory[25–27] that was created by Shannon is such mathematic tool that meets those requirements, and related theory comes directly from the physics underlying molecular binding interactions. In many researches, information theory play an import role in quantifying the sequence conservation in protein sequences and nucleotide.[28–35] The information content (IC) which is an import concept in the information theory will be used in the task of candidate TFBS searching to provide to assist in quantitative analysis[36]. Since the total information content of a model represents the distinguishable capability of the given model between a binding site (represented by the matrix) and the background model, therefore the information content of the given model in gene database should be fixed to a series of specified information content or fit some relationship in the experiment of quantitative analysis[37]. However, searching those models that meet those requirements is almost impossible in larger gene databases, so we generate a series of model meeting those requirements based on the theory of information content. We also make some contrast experiments on real-world datasets to show the universality of FLS algorithm. Related real-world and artificial datasets can be described as

- Datasets of Artificial Sequence SEQ1: the most sequences in the gene database usually are non-uniform background distribution. For contrast with sequences in gene databases, we generate some artificial sequences with approximate uniform background distribution to contrast the performance of three algorithms. The generated sequence is consisted of 55 megabases amino acid that every amino acid is randomly created and the corresponding background distribution is guaranteed to approximate to a uniform distribution.
- Datasets of Artificial Model MOD1: based on the theory of information content, Staden proposed an efficient method which can numerically estimate the probability-generating function for model with the given information content. When we get the probability-generating functions for each column of an alignment matrix, the probability-generating function for a multi-column alignment matrix[33] can be approximate replaced by the Staden’s approach. In order to contrast the influence of the different information content, we generate 26 matrixes which the length of all matrixes is fixed as 22 and information content of all matrix are increased gradually from 5 to 30. The single performance test is influenced seriously by the random error, so we generate 100 sets of the models using the same way and the averaged run time is viewed as the real performance of corresponding the algorithm.
- Datasets of Artificial Model MOD2: to contrast the influence of the different length of models on the performance of the three algorithms and eliminate the influence of the different information content of each model, the information content value of each model is set as 70% of the maximal information content of the corresponding model, and the lengths of all model are increased gradually from 5 to 30. We also generate 100 sets of the models using the same way and the averaged run time is approximately equal to the real performance of the corresponding algorithm.
- Datasets of Real-world Sequence SEQ2: to contrast with the artificial sequence, we concatenate to a 55 megabases long DNA sequences that all subsequences of DNA sequence are collected from the mouse and human genome.
- Datasets of Real-world Model MOD3: we collect 368 models about DNA from the known JASPAR database (JASPAR CORE REDUNANT 2016).[38, 39] the lengths of all models are also increased gradually from 5 to 21 and their average length is 13.2. In the real dataset, the number of models which their length exceed this range is very rare, so we left out specific modes. These models are divided into 25 groups which the model lengths in each group are increased from 5 to 21. In some group, due to the amount of some models having the same length are less than 25, so some models need to be repeated and divided into multiple groups at the same time.

### Time and accuracy performance

**Fig 2:**
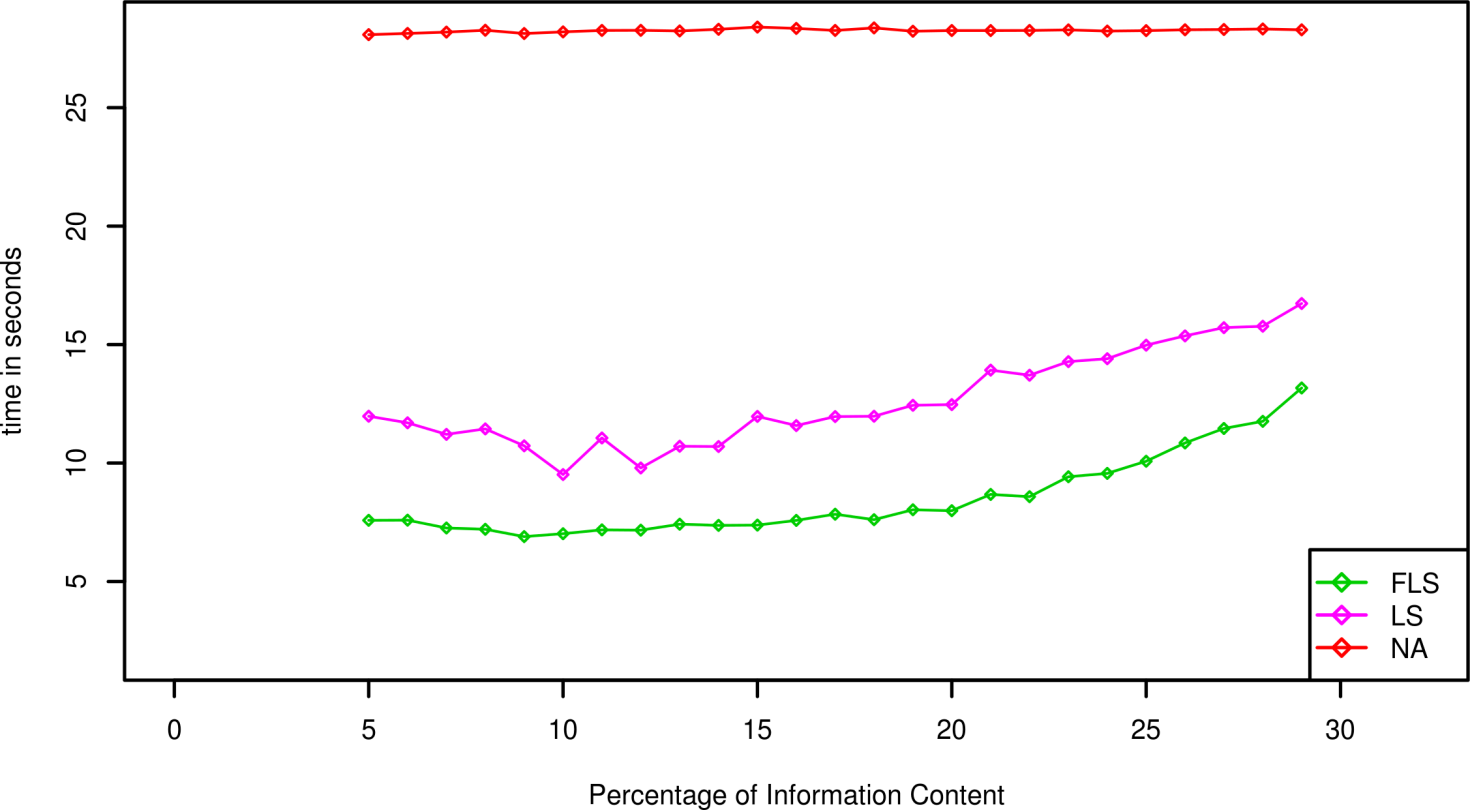
Average running times (in Seconds, Preprocessing excluded) of different algorithms for model MOD1 with p-value *γ* = 0.0001.

**Fig 3:**
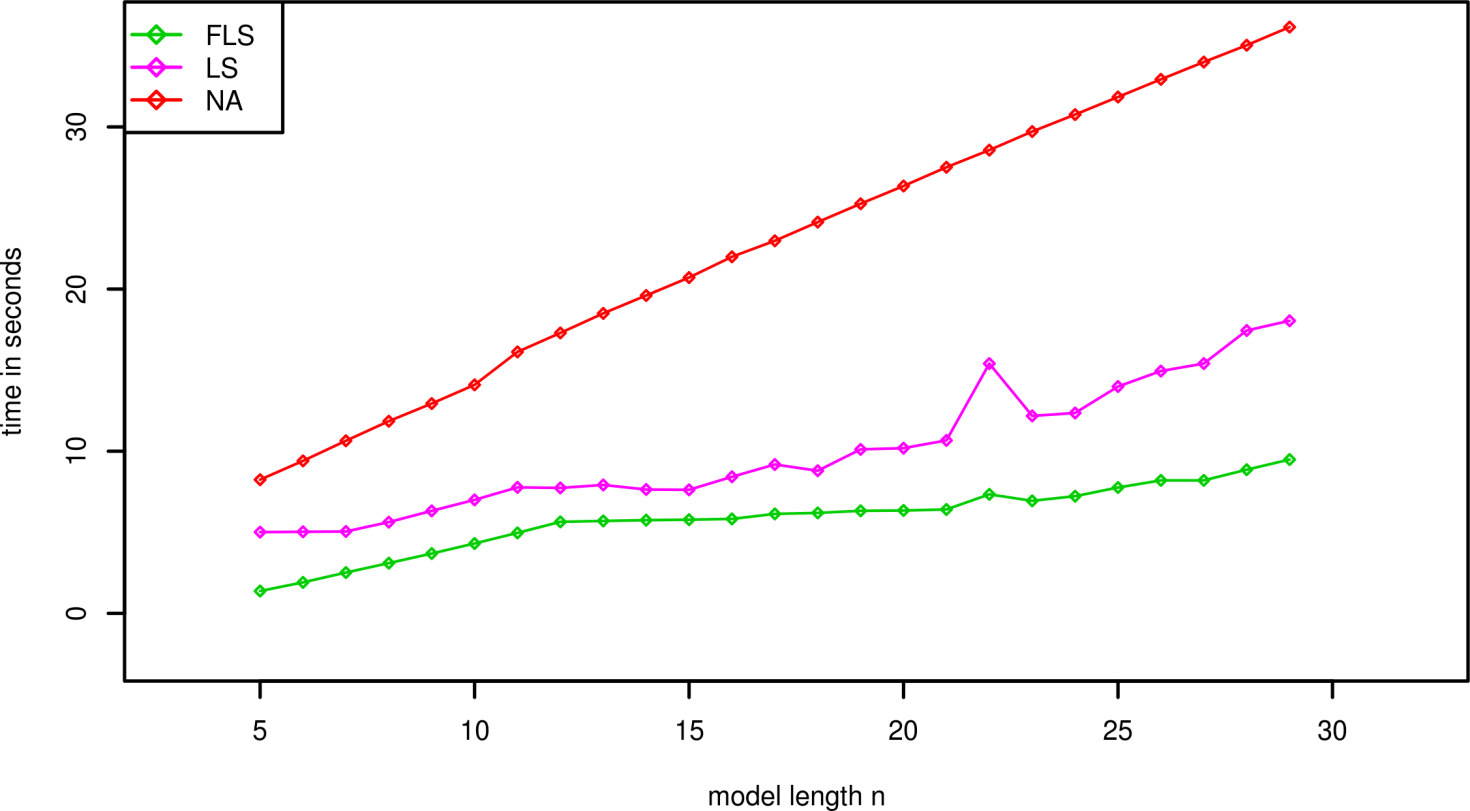
Average running times (in Seconds, Preprocessing excluded) of different algorithms for model MOD2 with p-value *γ* = 0.0001.

**Fig 4:**
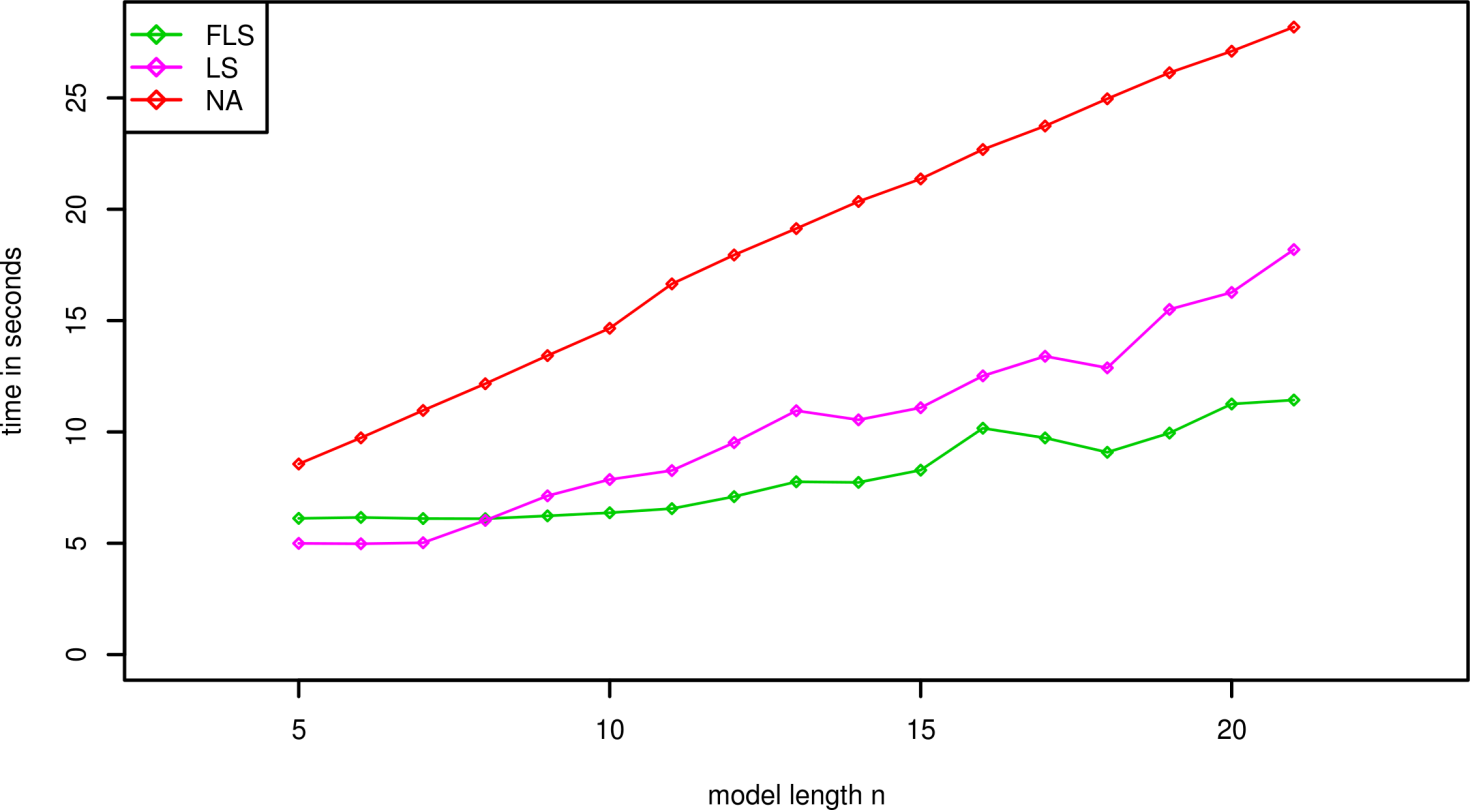
Average running times (in Seconds, Preprocessing excluded) of different algorithms for real-world model MOD3 and sequence SEQ2 with p-value *γ* = 0.0001.

The average running time on different artificial models or real-world model can be summarized in Figs 2–4. In those experiments, the p-values of all significant thresholds are set to the same value 0.0001.

The significant threshold *k* of each experiments is not given but is calculated indirectly through a given p-value. To evaluate performance of algorithms on varying significant thresholds, we have some experiments which the real-world models collect from JASPAR and the sequences are real-world SEQ2 with varying p-value. The average running times are summarized in Table 2. It’s obvious that the faster lookahead scoring algorithm outperforms others and has better performance with the smaller *γ*, on the other hand, the average running time of the naive algorithm almost be keep at 33

**Table 2:** Average running times of various p-value.

*γ* =	10^−1^	10^−2^	10^−3^	10^−4^	10^−5^	10^−6^
**NA**	34.83	32.53	32.48	32.99	33.85	34.02
**LS**	30.48	24.06	21.22	19.30	18.08	17.19
**FLS**	**22.39**	**16.43**	**13.26**	**11.67**	**9.92**	**9.32**

Average running times (in Seconds, Preprocessing excluded) of different algorithms for DNA pattern (*m* = 21) from JASPAR and varying p-Values, and each reported time is an average of 10 runs.

The various information content of models will produce different influence on the searching speed, so that we evaluate three algorithms on the artificial datasets contained the models MOD1 and the sequence SEQ1. The p-value is set to 0.0001 and the average running times are depicted as Fig 2. Obviously, the faster lookahead scoring algorithm is always the fastest searching one among three algorithms when the information content is same. Otherwise, the average running time also slow-growth when the information content of models increase gradually. There is a reasonable explanation for this phenomenon that the amount of the matching subsequence’s prefixes which their matching score is above the corresponding intermediate threshold will increase as containing more information content, such that the corresponding running time will also deteriorate.

The various length of models will produce different influence on the searching speed, so that we evaluate three algorithms on the artificial datasets contained models MOD2 and sequence SEQ1. The p-value is set to 0.0001 and the average running times are depicted as Fig 3. The faster lookahead scoring algorithm is still the fastest searching one among three contrastive algorithms. Although the average running times of three algorithms increase at the same time with the longer length of models, the running time of the faster lookahead scoring algorithm increases more slowly compared with the other algorithms.

To evaluate the performance of three algorithm on the real-world databases, we experimented on the dataset contained the models MOD3 and the sequence SEQ2. The p-value is also set to 0.0001 and the average running times are depicted as Fig 4. The faster lookahead scoring algorithm is still the fastest one with the other ones. Note that the average running time of FLS is more than LS’s one when the length of model is less than 8. The possible reason of this phenomenon can be described as that the advantage of permuted order of match will be disadvantage by adding additional operations compared with the LS algorithm when the length of model is too short.

## Conclusions

It should be emphasized that the results of all algorithms in above experiments are same so that we ignore contrast of results and all algorithms in our experiments can find out the precise result. Moreover, the faster lookahead scoring algorithm has a clear speed-up advantage compared with the lookahead searching algorithm. Through above contrasting experiments, the searching performance of FLS is better for dealing with real-world datasets and artificial datasets. As the exponential growth of both DNA and protein sequence databases, the searching speed of the faster lookahead scoring algorithm will be more significance and more concerned.
